# Serum Folate Forms Show Dose-Dependent Increases in Nonpregnant Ethiopian Women of Reproductive Age Participating in a Randomized Controlled Trial with Double-Fortified Salt Containing Iodine and Folic Acid

**DOI:** 10.1016/j.cdnut.2026.109398

**Published:** 2026-06-15

**Authors:** Christine M Pfeiffer, Zia Fazili, Charles D Arnold, Meseret Woldeyohannes, Debritu Nane, Mengistu Fereja, Masresha Tessema, Isaac Agbemafle, Homero Martinez, Christine M McDonald, Kenneth H Brown

**Affiliations:** 1 National Center for Environmental Health, Centers for Disease Control and Prevention, Atlanta, GA, United States; 2 Department of Nutrition and Institute for Global Nutrition, University of California, Davis, CA, United States; 3 Nutrition, Environmental Health, and Non-Communicable Disease Research Directorate, Ethiopian Public Health Institute, Addis Ababa, Ethiopia; 4 Department of Nutrition, University of Rhode Island, Kingston, RI, United States; 5 Research and Development Unit, Nutrition International, Ottawa, ON, Canada; 6 Department of Pediatrics, Division of Gastroenterology, Hepatology and Nutrition, University of California San Francisco, San Francisco, CA, United States

**Keywords:** 5-methyltetrahydrofolate, unmetabolized folic acid, nonmethyl folate, MeFox, total folate, HPLC-MS/MS, folic acid fortification, folate status

## Abstract

Information is limited regarding the response of serum folate forms (FOL-forms) to intake of folic acid (FA)–fortified foods. A randomized, 3-arm, dose–response trial of nonpregnant Ethiopian females assessed the effect of double-fortified salt containing iodine and FA on folate status. This report focuses on changes in FOL-forms {5-methyltetrahydrofolate (5-methylTHF), unmetabolized FA (UMFA), nonmethyl folate (NMFOL; sum of 3 minor FOL-forms), and the oxidation product of 5-methylTHF [pyrazino-s-triazine derivative of 4α-hydroxy-5-methyltetrahydrofolate (MeFox)]} after consumption of iodized salt (IS), lower FA IS (LFS; ∼200 μg additional FA/d), or higher FA IS (HFS; ∼600 μg additional FA/d) from baseline to endline ∼6 mo later. At endline, median concentrations of 5-methylTHF, UMFA, NMFOL, and MeFox were significantly different among treatment arms in a dose–response manner, whereas no differences were observed at baseline [5-methylTHF: 5.1-fold (HFS), 3.2-fold (LFS) baseline to endline increase]. The response of serum FOL-forms to the intervention was consistent with limited information from previous supplementation trials.

This trial was registered at clinicaltrials.gov as NCT06223854; https://clinicaltrials.gov/study/NCT06223854?term=NCT06223854&viewType=Card&rank=1.

## Introduction

Folate deficiency causes megaloblastic anemia; insufficiency increases the risk of neural tube birth defects [1]. Serum folate reflects short-term status. Most methods [e.g., microbiologic assay (MBA), protein-binding assays] measure total folate (TFOL) [2]. The assessment of individual folate forms (FOL-forms) by HPLC-MS/MS is technically complex but may elucidate associations with health outcomes and provide information on physiologic shifts in folate metabolism beyond TFOL. Folate, a water-soluble vitamin, comprises a family of vitamers with different metabolic functions: methyl-folate is involved in methylation reactions, and nonmethyl-folates (NMFOL) serve as cofactors for DNA synthesis [1]. FOL-forms have been studied in pregnancy and relative to colorectal adenoma risk and tumors, diabetes, and reduced renal function [[3], [4], [5], [6], [7], [8]]. They have been measured in the United Kingdom National Diet and Nutrition Survey, in response to folic acid (FA)–fortified wheat flour in the United States NHANES and in Cameroon and in response to supplementation with FA and/or 5-methyltetrahydrofolate (5-methylTHF) in women of reproductive age (WRA) [[9], [10], [11], [12], [13], [14], [15]].

Investigators in Ethiopia, a country with an unusually high birth prevalence of neural tube defects, conducted a community-based, household-randomized, dose–response trial to assess whether double-fortified salt with iodine and FA (DFS-IoFA) improves folate status in nonpregnant WRA [16]. After a 6-mo intervention period, serum and red blood cell folate concentrations measured by MBA increased significantly in 2 FA-fortification arms, whereas concentrations remained unchanged in the iodine-fortification arm. This trial offered a unique opportunity to study the dose–response of serum FOL-forms to FA-fortified salt in 3 intervention arms at 3 time points and to examine the contribution of serum FOL-forms to TFOL.

## Methods

### Study design, participants, and field procedures

This masked, 3-arm, dose–response trial was randomized at the household level and conducted in the Oromia Region in Ethiopia [17]. Briefly, ∼120 nonpregnant WRA (18**–**49 y) were randomly assigned to each of 3 intervention arms: *1*) higher FA iodized salt (IS; HFS) fortified with ∼99 parts per million (ppm) FA (group HFS; ∼600 μg additional FA/d), *2*) lower FA IS (LFS) fortified with ∼33 ppm FA (group LFS; ∼200 μg additional FA/d), or *3*) IS with no added FA (group IS, control group) (Supplemental Figure 1). All salts were fortified with ∼32 ppm iodine as potassium iodate. Mean FA intakes (μg/d) from fortified salt were 788 ± 184 (HFS group) and 277 ± 66 (LFS group) [16]. For information on salt administration and compliance, see Supplemental Text 1.

Fasting venous blood was obtained at baseline, at a randomly assigned intermediate time between 4 and 20 wk, and at endline (24**–**26 wk after initiating the intervention). Whole blood vacutainers were allowed to coagulate for 30 min before centrifugation at 3000 × *g*. Serum aliquots were stored at –20°C for ≤1 wk at the field site, then transferred to the Ethiopian Public Health Institute laboratory for storage at <–80°C until dry ice shipment to the Centers for Disease Control and Prevention (CDC) for analysis [17].

### Measurement of serum FOL-forms

We measured 5 biologically active serum FOL-forms by HPLC-MS/MS: 5-methylTHF, unmetabolized FA (UMFA), 5-formyltetrahydrofolate, tetrahydrofolate (THF), and 5,10-methenyltetrahydrofolate [18]. We calculated the sum of the latter 3 forms as NMFOL because concentrations are typically less than the limit of detection (LOD) and can be a result of folate interconversions at slightly acidic pH during sample preparation [19]. NMFOL is less than the LOD if all 3 forms are less than the LOD, and is greater than or equal to LOD if ≥1 form is greater than or equal to LOD. We also measured the pyrazino-s-triazine derivative of 4α-hydroxy-5-methylTHF (MeFox), a biologically inactive oxidation product of 5-methylTHF. We calculated serum TFOL as the sum of the 5 biologically active forms, using imputed values (LOD / √2) for any FOL-form result less than the LOD. No TFOL was calculated when ≥1 FOL-form result was missing (10 samples). For method performance information, see Supplemental Text 1.

### Statistical analysis

The distributions of serum FOL-forms are log-normal. We, therefore, reported median and IQR concentrations and used analysis of covariance models with log-transformed outcomes to estimate geometric mean ratios for group-wise comparisons. We fit the models using data from all 3 intervention arms and calculated a global *P* value for each folate outcome variable and time point to assess overall differences across intervention arms. Models for the intermediate and endline time point controlled for the baseline measure. We then assessed cross-sectional intervention effects based on post hoc pairwise comparisons using multiplicity-adjusted *P* values (Hommel adjustment). We also assessed changes from baseline to endline within each intervention arm visually with fitted locally estimated scatterplot smoothing (LOESS) curves and conducted paired *t*-tests on log-transformed outcomes. Data management and statistical analyses were conducted using Stata v19.5 and R v4.43 [20,21]. The Ethiopian Public Health Institute Institutional Review Board approved the research protocol. CDC exempted the protocol from full review as investigators received deidentified data.

## Results

### Serum FOL-form concentrations by intervention arm and time point

At baseline, intermediate, and endline time points, serum FOL-forms data were available for 113**–**125, 101**–**108, and 103**–**108 WRA per intervention arm, respectively (Table 1). No statistically significant differences in dropout rates were observed between the study arms after adjustment for multiple comparisons. At baseline, the overall (all 3 intervention arms) median TFOL and 5-methylTHF concentrations were 13.8 and 12.8 nmol/L, respectively; the overall median UMFA, NMFOL, and MeFox concentrations were 0.19, 0.61, and 1.37 nmol/L, respectively; 31%, 42%, and 0% of participants had concentrations less than the LOD, respectively (data not shown). Supplemental Table 1 shows descriptive data [mean (SD), range] for serum TFOL and FOL-form concentrations.

**TABLE 1:** Concentrations of serum TFOL and folate forms by intervention arm and time point, percent of TFOL for serum folate forms, and sample sizes

Biomarker	Time point^2^	Intervention arm^1^			Global
		HFS	LFS	IS	*P* value^3^
Median (IQR) concentrations (nmol/L)					
TFOL	Baseline	13.2^a^ (9.79, 17.6)	14.8^a^ (11.2, 19.2)	13.3^a^ (10.1, 17.9)	0.149
	Intermediate	54.9^a^ (43.3, 66.8)	37.8^b^ (24.2, 49.1)	12.5^c^ (10.8, 15.7)	<0.001
	Endline	65.0^a^ (56.6, 74.2)	45.5^b^ (36.6, 59.5)	14.2^c^ (10.7, 18.3)	<0.001
	*P* value^4^	<0.001	<0.001	0.070	
5-MethylTHF	Baseline	12.3^a^ (8.77, 16.8)	13.7^a^ (10.2, 18.0)	12.4^a^ (9.43, 16.7)	0.171
	Intermediate	53.0^a^ (41.5, 65.3)	36.2^b^ (22.9, 47.6)	11.6^c^ (10.0, 14.9)	<0.001
	Endline	62.8^a^ (54.3, 72.0)	44.2^b^ (34.4, 57.7)	13.3^c^ (9.93, 17.5)	<0.001
	*P* value^4^	<0.001	<0.001	0.049	
UMFA	Baseline	0.19^a^ (0.10, 0.27)	0.20^a^ (0.10, 0.28)	0.18^a^ (0.10, 0.25)	0.181
	Intermediate	1.25^a^ (0.96, 1.59)	0.89^b^ (0.67, 1.08)	0.18^c^ (0.10, 0.29)	<0.001
	Endline	1.24^a^ (0.92, 1.71)	0.85^b^ (0.72, 1.07)	0.17^c^ (0.10, 0.24)	<0.001
	*P* value^4^	<0.001	<0.001	0.958	
NMFOL	Baseline	0.55^a^ (0.46, 0.82)	0.64^a^ (0.46, 0.85)	0.64^a^ (0.46, 0.87)	0.324
	Intermediate	0.46^a^ (0.46, 0.75)	0.46^a^ (0.46, 0.68)	0.46^a^ (0.46, 0.67)	0.282
	Endline	0.77^a^ (0.56, 0.99)	0.72^b^ (0.46, 0.90)	0.56^c^ (0.46, 0.73)	<0.001
	*P* value^4^	<0.001	0.341	0.022	
MeFox	Baseline	1.38^a^ (0.91, 2.01)	1.36^a^ (0.92, 2.27)	1.37^a^ (0.81, 2.28)	0.629
	Intermediate	1.95^a^ (1.26, 2.88)	1.35^b^ (1.00, 2.11)	0.98^c^ (0.66, 1.59)	<0.001
	Endline	2.26^a^ (1.70, 3.60)	1.56^b^ (1.23, 2.29)	1.11^c^ (0.68, 1.79)	<0.001
	*P* value^4^	<0.001	0.097	0.004	
Median percentage of TFOL (%)					
5-MethylTHF	Baseline	93.9^a^ (91.4, 95.2)	94.0^a^ (91.9, 95.6)	93.4^a^ (91.9, 95.2)	0.827
	Intermediate	96.5^a^ (95.5, 97.3)	95.7^b^ (94.6, 96.9)	94.2^c^ (91.8, 95.4)	<0.001
	Endline	96.7^a^ (95.9, 97.4)	96.5^a^ (95.3, 97.4)	94.3^b^ (92.7, 95.7)	<0.001
UMFA	Baseline	1.31^a^ (0.97, 2.26)	1.37^a^ (0.91, 2.02)	1.39^a^ (0.89, 1.89)	0.661
	Intermediate	2.26^a^ (1.72, 2.98)	2.40^a^ (1.62, 3.27)	1.48^b^ (0.90, 2.27)	<0.001
	Endline	1.85^a^ (1.41, 2.67)	1.72^a^ (1.39, 2.59)	1.28^b^ (0.79, 1.87)	<0.001
NMFOL	Baseline	4.64^a^ (3.26, 5.90)	4.58^a^ (3.03, 5.53)	4.88^a^ (3.77, 6.65)	0.269
	Intermediate	1.07^a^ (0.78, 1.40)	1.64^b^ (1.13, 2.25)	4.14^c^ (3.48, 5.69)	<0.001
	Endline	1.21^a^ (0.99, 1.68)	1.52^b^ (1.03, 2.24)	4.45^c^ (3.02, 5.67)	<0.001
Sample sizes (*n*)^5^					
TFOL	Baseline	125	113	122	
	Intermediate	101	105	105	
	Endline	103	108	104	
5-MethylTHF	Baseline	125	113	122	
	Intermediate	102	106	107	
	Endline	105	108	105	
UMFA	Baseline	125	113	122	
	Intermediate	102	106	108	
	Endline	105	108	106	
NMFOL	Baseline	125	113	122	
	Intermediate	101	105	106	
	Endline	103	108	105	
MeFox	Baseline	125	113	122	
	Intermediate	102	106	108	
	Endline	105	108	106	

Abbreviations: 5-methylTHF, 5-methyltetrahydrofolate; HFS, higher folic acid iodized salt; IS, iodized salt with no added folic acid; LFS, lower folic acid iodized salt; MeFox, pyrazino-s-triazine derivative of 4α-hydroxy-5-methyltetrahydrofolate; NMFOL, nonmethyl folate; TFOL, total folate; UMFA, unmetabolized folic acid.

1 Different superscript letters (e.g., a, b, and c) designate significant differences from post hoc pairwise comparisons assessing cross-sectional intervention effects (using multiplicity-adjusted *P* values via the Hommel adjustment), i.e., comparing columns. Assessment at the intermediate and endline time points controls for baseline measures. All biomarkers were log-transformed for analysis. HFS fortified with ∼99 ppm folic acid to provide an additional 600 μg folic acid/d; LFS fortified with ∼33 ppm folic acid to provide an additional 200 μg folic acid/d; all salts fortified with ∼32 ppm iodine as potassium iodate.

2 Intermediate time point randomly assigned between 4 and 20 wk; endline at 24–26 wk after initiating the intervention.

3 Global *P* value assesses overall differences across the intervention arms for each folate outcome variable and time point in analysis of covariance models. Assessment at the intermediate and endline time points controls for the baseline measure. All biomarkers were log-transformed for analysis.

4 *P* value assesses changes from baseline to endline within each intervention arm. All were biomarkers log-transformed for analysis.

5 There are small sample size differences because for 2 samples, the laboratory could not generate a valid result for each folate form.

**FIGURE 1:**
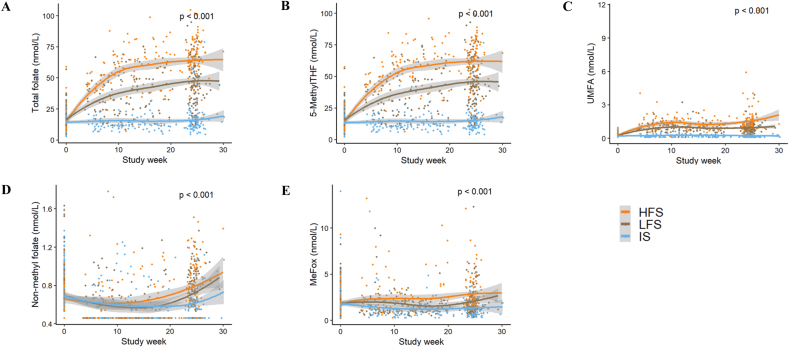
(A–E) Concentrations of serum total folate and folate forms by study week and intervention arm. The intermediate time point was randomly assigned between 4 and 20 wk. The endline was at 24–26 wk after initiating the intervention. The global *P* value assesses overall differences across the intervention arms for each folate outcome variable. 5-methylTHF, 5-methyltetrahydrofolate; HFS, higher folic acid iodized salt; IS, iodized salt with no added folic acid; LFS, lower folic acid iodized salt; MeFox, pyrazino-s-triazine derivative of 4α-hydroxy-5-methyltetrahydrofolate; UMFA, unmetabolized folic acid.

All folate outcome variables (5-methylTHF, UMFA, NMFOL, MeFox, and TFOL) showed statistically significant overall differences in concentrations (global *P* value) and post hoc pairwise comparison differences among the 3 intervention arms at the intermediate and endline time points (except NMFOL at the intermediate time point), but not at baseline (Table 1). After ∼6 mo of exposure to study salts, median folate concentrations increased significantly in groups HFS and LFS (except NMFOL in the LFS group) but stayed similar in group IS. 5-MethylTHF and UMFA concentrations increased ∼5- and 6-fold (HFS group) and 3- and 4-fold (LFS group), respectively, whereas NMFOL and MeFox concentrations increased ∼1.4- and 1.6-fold (HFS group) and 1.1- and 1.1-fold (LFS group), respectively. The response to intervention can also be observed by a right shift in the distributions (Supplemental Figure 2). Mean TFOL (panel A), 5-methylTHF (panel B), and UMFA (panel C) concentrations showed a nonlinear increase, rapid during the first 12 wk and slower thereafter; mean NMFOL (panel D) and MeFox (panel E) concentrations changed less (Figure 1).

### Contribution of FOL-forms to serum TFOL

At baseline, the overall (all 3 intervention arms) contribution of 5-methylTHF, UMFA, and NMFOL to TFOL was ∼94%, 1.4%, and 4.7%, respectively (data not shown). Supplemental Table 1 shows the mean (SD) and range contribution of FOL-forms to TFOL. There was no difference among the 3 intervention arms (Table 1). At endline, the contribution of 5-methylTHF (96.7% in HFS and 96.5% in LFS group) and UMFA (1.85% in HFS and 1.72% in LFS group) increased slightly, whereas the contribution of NMFOL (1.21% in HFS and 1.52% in LFS group) decreased. All pairwise differences between the HFS and IS and LFS and IS groups were significant at the intermediate and endline time points.

### Correlations between various serum folate biomarkers

The Spearman correlations between 5-methylTHF and TFOL and between THF and NMFOL were high (*r >*0.99) regardless of the intervention arm or time point (Supplemental Table 2). Other correlations were mostly weak (*r* ≤0.4) and not significant.

## Discussion

The randomized controlled trial of DFS-IoFA in Ethiopia provides novel data for serum FOL-forms before and after a fortification intervention. We noted a clear dose–response increase of FOL-forms in WRA consuming DFS-IoFA compared with those consuming IS, with pronounced effects for 5-methylTHF and UMFA and subtle distribution shifts for NMFOL and MeFox.

5-MethylTHF concentrations stabilized in both intervention arms by 6 mo. UMFA concentrations stabilized in the LFS arm but likely did not reach equilibrium in the HFS arm (slope of concentration-by-time curve >0). Serum folate (by MBA) stabilized by ∼week 20 [16], suggesting a slight delay in the UMFA response. No study participant had a higher UMFA contribution to TFOL than 15.4% (HFS group at endline), and the highest postintervention UMFA concentrations were 4.05 (LFS group) and 11.5 (HFS group) nmol/L (Supplemental Table 1), with nearly 80% of UMFA concentrations ≤1 nmol/L. Although questions have been raised about high FA intakes and the increase in serum UMFA in response to FA fortification, multiple expert committees have concluded that there are no confirmed adverse effects [[22], [23], [24], [25]].

The small increase of NMFOL during the later part of the intervention is due to THF, as 99% of samples had 5-formyltetrahydrofolate and 5,10-methenyltetrahydrofolate concentrations less than the LOD. At endline, the median (IQR) THF concentration (nmol/L) was higher in the LFS [0.44 (0.18, 0.62); *P* = 0.002] and HFS [0.47 (0.27, 0.70); *P* = 0.002] compared with the IS [0.27 (0.18, 0.44)] group. However, we found no significant correlation between THF and UMFA at either time point or intervention arm. Thus, the small postintervention increase in THF is probably not a saturation effect where extra FA intake appears in circulation as UMFA or THF.

We compared responses of FOL-forms in this study to available data from cross-sectional or randomized controlled FA-intervention studies (Supplemental Table 3). Participants in the LFS group consumed an additional 277 μg/d FA for 6 mo. At endline, their serum FOL-forms concentrations were comparable to NHANES 2011**–**2016 data from United States females aged ≥1 y [11] who were estimated to consume an additional 100**–**200 μg/d FA [26]. The LFS group also showed similar concentrations to plasma FOL-forms from Cameroonian females 1 y after the introduction of mandatory wheat flour fortification [12]. The lower 5-methylTHF and higher MeFox concentrations in the Cameroon samples likely reflect a 5-methylTHF loss due to oxidation during sample handling, shown to occur in EDTA plasma samples [18]. The CDC HPLC-MS/MS method does not generate MeFox beyond what is present at blood collection [27]. The decrease in the MeFox/5-methylTHF ratio from baseline (∼0.1) to postintervention (∼0.035) indicates that MeFox likely occurs in vivo rather than being formed in vitro as a proportion of 5-methylTHF, as hypothesized previously based on different associations with lifestyle and physiologic factors between 5-methylTHF and MeFox [[10], [11]].

Honduran females exposed to oral FA intake of 1000 μg/d for 12 wk [28] showed similar 5-methylTHF but higher UMFA concentrations [14] compared with the current study. The higher UMFA may be due to the higher FA dose and/or the implementation of a wheat flour fortification program in Central American countries in 2002 (1.8 mg FA/kg flour), a few years before the Honduran supplementation trial. Chinese females exposed to oral FA intake of 100, 400, or 4000 μg/d for 6 mo [29] showed lower initial 5-methylTHF concentrations but similar dose–response and similar or lower UMFA concentrations [13] compared with the current study. The low UMFA concentrations in response to the 4000 μg/d dose may be due to urinary folate excretion, given the body’s limited ability to absorb and reduce large FA doses [1].

The strengths of this study are: *1*) its randomized design with 2 levels of FA fortification and a control group, *2*) the variable intermediate time point allowing the examination of folate biomarker response kinetics, *3*) an adequate sample size to detect differences in key outcomes, and *4*) the unique, high-quality data for serum FOL-forms. Because the trial was conducted in generally healthy WRA, it is unclear whether the dose–response findings and/or the distribution of FOL-forms are applicable to other age or ethnic groups or to individuals with chronic conditions. In summary, the response of serum FOL-forms in the Ethiopian intervention trial was consistent with limited information from supplementation trials. The TFOL response was comparable to that of serum and red blood cell folate measured by MBA [16], further strengthening the findings. The low postintervention UMFA concentrations can reassure public health officials planning population-wide FA fortification.

## Author contributions

The authors’ responsibilities were as follows – CMP, KHB: designed the research project; CMP, ZF: conducted the research; CDA: analyzed the data; CMP: wrote the paper and took primary responsibility for the final content; ZF, CDA, MW, DN, MF, MT, IA, HM, CMM, KHB: took responsibility for the design of the parent trial and related data collection and specimen processing; and all authors: read and approved the final manuscript.

## Data availability

The study protocol (12) and statistical analysis plan (https://osf.io/pu537/) for the parent trial are publicly available. Final databases with variable names and definitions and deidentified individual participant information are archived at the Ethiopian National Data Management Center at EPHI (https://ndmc.ephi.gov.et/) and will be made available to scientists from other institutions following publication. The metadata are publicly available at https://doi.org/10.7910/DVN/JZCF2N. Individuals who request access to the data set will be required to describe in writing the objectives of their analyses, the variables requested, and any plans for collaboration with the core research team.

## Disclosure

The findings and conclusions in this report are those of the authors and do not necessarily represent the official views or positions of the Centers for Disease Control and Prevention or the Department of Health and Human Services.

## Declaration of generative AI and AI-assisted technologies in the writing process

The authors declare that no generative AI or AI-assisted technologies were used in the writing of this manuscript.

## Funding

The intervention trial and sample collection were carried out with the support of a grant from Nutrition International, Ottawa, Canada, through the financial assistance of the Gates Foundation.

## Conflict of interest

The authors report no conflicts of interest.

## References

[1] Bailey L.B., Stover P.J., McNulty H., Fenech M.F., Gregory J.F., Mills J.L. (2015). Biomarkers of nutrition for development—folate review. J. Nutr..

[2] Pfeiffer C.M., Fazili Z., Zhang M., Bailey L.B. (2010). Folate in Health and Disease.

[3] Obeid R., Kasoha M., Kirsch S.H., Munz W., Herrman W. (2010). Concentrations of unmetabolized folic acid and primary folate forms in pregnant women at delivery and in umbilical cord blood. Am. J. Clin. Nutr..

[4] Cochrane K.M., Elango R., Devlin A.M., Mayer C., Hutcheon J.A., Karakochuk C.D. (2024). Supplementation with (6S)-5-methyltetrahydrofolic acid appears as effective as folic acid in maintaining maternal folate status while reducing unmetabolized folic acid in maternal plasma: a randomized trial of pregnant women in Canada. Br. J. Nutr..

[5] Rees J.R., Morris C.B., Peacock J.L., Ueland P.M., Barry E.L., McKeown-Eyssen G.E. (2017). Unmetabolized folic acid, tetrahydrofolate and colorectal adenoma risk. Cancer Prev. Res. (Phila)..

[6] Liu J., Hesson L.B., Meagher A.P., Bourke M.J., Hawkins N.J., Rand K.N. (2012). Relative distribution of folate species is associated with global DNA methylation in human colorectal mucosa. Cancer Prev. Res. (Phila)..

[7] Crider K.S., Adisa O., Pfeiffer C.M., Wang A., Zhou Y., Yeung L.F. (2026). Prediabetes, diabetes, and folate status among United States women of reproductive age: NHANES 2011–March 2020. Am. J. Clin. Nutr..

[8] Wang A., Yeung L.F., Burrows N.R., Rose C.E., Fazili Z., Pfeiffer C.M. (2022). Reduced kidney function is associated with increasing red blood cell folate concentration and changes in folate form distributions (NHANES 2011–2018). Nutrients.

[9] Jones K.S., Collins D., Meadows S.R., Koulman A., Page P. (2023). National Diet and Nutrition Survey data reveal a decline in folate status in the United Kingdom population between 2008 and 2019. Am. J. Clin. Nutr..

[10] Pfeiffer C.M., Sternberg M.R., Fazili Z., Lacher D.A., Zhang M., Johnson C.L. (2015). Folate status and concentrations of serum folate forms in the US population: National Health and Nutrition Examination Survey 2011–2. Br. J. Nutr..

[11] Fazili Z., Sternberg M.R., Potischman N., Wang C.Y., Storandt R.J., Yeung L. (2020). Demographic, physiologic, and lifestyle characteristics observed with serum total folate differ among folate forms: cross-sectional data from fasting samples in the NHANES 2011-2016. J. Nutr..

[12] Engle-Stone R., Nankap M., Ndjebayi A.O., Allen L.H., Shahab-Ferdows S., Hampel D. (2017). Iron, zinc, folate, and vitamin B-12 status increased among women and children in Yaoundé and Douala, Cameroon, 1 year after introducing fortified wheat flour. J. Nutr..

[13] Pfeiffer C.M., Fazili Z., Hao L., Berry R.J., Bailey L.B., Zhu J. (2008). Folic acid dose, MTHFR C677T genotype, and temporal effects on plasma folate species. FASEB J.

[14] Fazili Z., Paladugula N., Rosenthal J., Crista K., Pfeiffer C.M. (2011). Does folic acid accumulate in serum with a daily dose of 1 mg administered over 12 weeks?. FASEB. J..

[15] Obeid R., Rube E., Schӧn C., Geisel J. (2024). Serum concentrations of folate forms following supplementation of multimicronutrients with 400 μg or 800 μg mix of (6S)-5-methyltetrahydrofolate and folic acid (1:1) in women of childbearing age. Mol. Nutr. Food. Res..

[16] Tessema M., Nane D., Woldeyohannes M., Agbemafle I., McDonald C.M., Arnold C.D. (2026). Folic acid fortification of iodized salt improves the folate status of nonpregnant adult Ethiopian females and does not impair their iodine status: a community-based, household-randomized, dose-response trial. Am. J. Clin. Nutr..

[17] Brown K.H., Tessema M., McDonald C.M., Agbemafle I., Wodeyohannes M., Fereja M. (2024). Protocol for a community-based, household-randomised, dose-response trial to assess the acceptability, nutritional effects and safety of double-fortified salt containing iodine and folic acid compared with iodised salt among non-pregnant Ethiopian women of reproductive age (DFS-IoFA). BMJ Open.

[18] Fazili Z., Whitehead R.D., Paladugula N., Pfeiffer C.M. (2013). A high-throughput LC-MS/MS method suitable for population biomonitoring measures five serum folate vitamers and one oxidation product. Anal. Bioanal. Chem..

[19] Pfeiffer C.M., Fazili Z., McCoy L., Zhang M., Gunter E.W. (2004). Determination of folate vitamers in human serum by stable-isotope dilution tandem mass spectrometry and comparison to radioassay and microbiologic assay. Clin. Chem..

[20] StataCorp (2005). http://www.stata.com.

[21] R Core Team, R (2025). https://www.r-project.org/.

[22] National Toxicology Program (2015). https://ntp.niehs.nih.gov/ntp/ohat/folicacid/final_monograph_508.pdf.

[23] Public Health England (2017). https://assets.publishing.service.gov.uk/media/5a82c5e240f0b62305b94444/SACN_Update_on_folic_acid.pdf.

[24] Field M.S., Stover P.J. (2018). Safety of folic acid. Ann. N.Y. Acad. Sci..

[25] Maruvada P., Stover P.J., Mason J.B., Bailey R.L., Davis C.D., Field M.S. (2020). Knowledge gaps in understanding the metabolic and clinical effects of excess folates/folic acid: a summary, and perspectives, from an NIH workshop. Am. J. Clin. Nutr..

[26] Crider K.S., Bailey L.B., Berry R.J. (2011). Folic acid fortification—its history, effect, concerns, and future directions. Nutrients.

[27] Fazili Z., Pfeiffer C.M. (2013). Accounting for an isobaric interference allows correct determination of folate vitamers in serum by isotope dilution-liquid chromatography-tandem MS. J. Nutr..

[28] Rosenthal J., Milla G., Flores A., Yon M., Pfeiffer C., Umaña E. (2008). Effect of different dosage and administration schedules of folic acid on blood folate levels in a population of Honduran women of reproductive age. Public Health. Nutr..

[29] Hao L., Yang Q.H., Li Z., Bailey L.B., Zhu J.-H., Hu D.J. (2008). Folate status and homocysteine response to folic acid doses and withdrawal among young Chinese women in a large-scale randomized double-blind trial. Am. J. Clin. Nutr..

