# Supplementary material for: Serum Folate Forms Show Dose-Dependent Increases in Nonpregnant Ethiopian Women of Reproductive Age Participating in a Randomized Controlled Trial with Double-Fortified Salt Containing Iodine and Folic Acid
Source: Curr Dev Nutr. 2026 Jun 15;10(7):109398. doi: 10.1016/j.cdnut.2026.109398 (PMC13355729; doi:10.1016/j.cdnut.2026.109398)
Supplement: Multimedia component 1 [file mmc1.docx]

**Title: Serum folate forms show dose-dependent increases in non-pregnant Ethiopian women of reproductive age participating in a randomized controlled trial with double-fortified salt containing iodine and folic acid**

**First author: Pfeiffer CM**

**Supplementary Text 1.**

**Information on salt administration and compliance.**

Tessema et al. (**1**) provide detailed information on salt administration and compliance. Briefly, salt was delivered every 2 wk for 26 wk. At the time of delivery, leftover salt from the previous delivery was collected, weighed, and removed from the household. The distributors repeatedly advised participants to use only the study salt for all cooking and seasoning, but not for animal feeds or sharing with neighbors. The mean household salt disappearance per capita was 13.1±5.9 g/d and did not differ by intervention arm. The participants’ mean usual discretionary salt intake during the trial period, as measured by weighed food records, was 8.4±1.7 g/d, of which 7.8±1.8 g/d (94%) was the study salt. The greater daily household salt disappearance rate per capita compared to the participants’ observed intakes was likely because some salt is discarded along with food waste, sizable amounts of salt are used intermittently to prepare salt-containing spice mixtures, but relatively small amounts of these spice mixtures are used daily, and the mean per capita household salt disappearance rate included salt consumed by adult males in the household, whose intakes are greater than those of adult females.

The consumption of study salts and other salts did not differ by intervention arm. The overall median urinary iodine/creatinine ratio during the intervention was 39.1 (28.3, 54.3) μg/mmol, which was greater than preintervention, and there were no differences by intervention arm. While the FA-fortified salt had a faint yellow hue, this did not reduce its acceptability. FA-fortification neither affected household salt disappearance nor salt consumption by study participants.

**Method performance information for measurement of serum folate forms.**

LOD values (nmol/L) for the folate forms were: 5-methylTHF, 0.13; UMFA, 0.14; 5-formyl-tetrahydrofolate, 0.20; tetrahydrofolate, 0.25; 5,10-methenyltetrahydrofolate, 0.20; and MeFox, 0.10. Coefficients of variation for 2**–**3 QC pools analyzed across nearly 40 study runs were: 2.9%**–**3.8% for 5-methylTHF, 3.9%**–**9.0% for UMFA, 5.7%**–**6.0% for MeFox, and 3.5%**–**6.6% for the 3 minor forms making up NMFOL. We analyzed 10 blind QC pools at a rate of 1 blind QC sample in every 20 study samples (each blind QC pool was measured 5**–**9 times across the study runs) and achieved the following mean relative difference to the target value: <±3.4% for 5-methylTHF, <±7.3% for UMFA, and <±4.2% for MeFox.

Abbreviations: 5-methylTHF, 5-methyltetrahydrofolate; MeFox, pyrazino-s-triazine derivative of 4α-hydroxy-5-methyltetrahydrofolate; NMFOL, non-methyl folate; QC, quality control; UMFA, unmetabolized folic acid.

**Reference**

1. M. Tessema, D. Nane, M. Woldeyohannes, I. Agbemafle, C.M. McDonald, C.D. Arnold, *et al.*, Folic acid fortification of iodized salt improves the folate status of nonpregnant adult Ethiopian females and does not impair their iodine status: a community-based, household-randomized, dose-response trial, Am. J. Clin. Nutr. 123 (2026) 101204, <https://doi.org/10.1016/j.ajcnut.2026.101204>.

**Supplementary Table 1.** Mean and range of concentrations for serum total folate and folate forms by intervention arm and timepoint ^1^

|  |  | **Intervention arm^2^** | | | | | |
| --- | --- | --- | --- | --- | --- | --- | --- |
| **Biomarker** | **Timepoint^3^** | **HFS** | | **LFS** | | **IS** | |
|  |  | **Mean (SD)** | **Range** | **Mean (SD)** | **Range** | **Mean (SD)** | **Range** |
| ***Concentration (nmol/L)*** | |  |  |  |  |  |  |
| TFOL | Baseline | 14.8 (7.65) | 3.39–58.9 | 16.1 (7.70) | 4.77–57.0 | 14.3 (5.82) | 2.80–32.5 |
|  | Intermediate | 54.7 (17.9) | 7.11– 8.8 | 37.7 (16.6) | 8.44–75.4 | 14.7 (8.6) | 4.85–63.8 |
|  | Endline | 63.8 (17.8) | 15.4–105 | 47.9 (17.5) | 12.4–94.8 | 16.2 (10.5) | 4.52–78.9 |
| 5-MethylTHF | Baseline | 13.9 (7.61) | 2.51–57.6 | 15.1 (7.68) | 3.78–56.4 | 13.4 (5.74) | 2.20–31.4 |
|  | Intermediate | 52.8 (17.6) | 6.24–95.8 | 36.2 (47.6) | 7.88–74.3 | 13.8 (8.37) | 4.29–61.3 |
|  | Endline | 61.6 (17.5) | 14.0–103 | 46.3 (17.4) | 11.5–92.8 | 15.4 (10.2) | 3.96–76.6 |
| UMFA | Baseline | 0.22 (0.14) | 0.10–0.79 | 0.23 (0.15) | 0.10–0.92 | 0.20 (0.12) | 0.10–0.18 |
|  | Intermediate | 1.32 (0.59) | 0.10–4.05 | 0.92 (0.46) | 0.10–3.23 | 0.24 (0.22) | 0.10–0.18 |
|  | Endline | 1.52 (1.31) | 0.20–11.5 | 0.93 (0.44) | 0.10–3.90 | 0.22 (0.19) | 0.10–0.17 |
| NMFOL | Baseline | 0.66 (0.25) | 0.46–1.59 | 0.70 (0.27) | 0.46–1.63 | 0.70 (0.26) | 0.46–1.58 |
|  | Intermediate | 0.63 (0.27) | 0.46–1.78 | 0.59 (0.19) | 0.46–1.27 | 0.59 (0.20) | 0.46–1.25 |
|  | Endline | 0.80 (0.27) | 0.46–1.51 | 0.72 (0.24) | 0.46–1.37 | 0.61 (0.18) | 0.46–1.12 |
| MeFox | Baseline | 1.73 (1.34) | 0.25–9.31 | 1.86 (1.43) | 0.27–8.25 | 1.80 (1.74) | 0.23–14.0 |
|  | Intermediate | 2.54 (2.12) | 0.30–13.2 | 1.77 (1.44) | 0.50–9.97 | 1.32 (1.34) | 0.28–11.8 |
|  | Endline | 2.81 (1.87) | 0.51–12.1 | 2.06 (1.60) | 0.34–12.3 | 1.36 (0.94) | 0.30–12.3 |
| ***Percent of TFOL (%)*** | |  |  |  |  |  |  |
| 5-MethylTHF | Baseline | 92.9 (3.75) | 74.0–98.7 | 93.1 (3.69) | 79.3–98.9 | 92.9 (3.33) | 78.6–97.6 |
|  | Intermediate | 96.1 (1.77) | 87.7–98.3 | 95.2 (2.42) | 85.6–98.5 | 93.6 (2.64) | 85.6–98.3 |
|  | Endline | 96.2 (2.11) | 84.0–98.4 | 96.1 (1.77) | 90.7–98.4 | 94.0 (2.31) | 86.9–98.1 |
| UMFA | Baseline | 1.77 (1.21) | 0.24–6.40 | 1.66 (1.17) | 0.26–7.37 | 1.57 (0.98) | 0.37–5.45 |
|  | Intermediate | 2.56 (1.27) | 0.64–8.80 | 2.81 (1.83) | 0.49–11.2 | 1.70 (0.99) | 0.41–5.02 |
|  | Endline | 2.46 (1.97) | 0.54–15.4 | 2.16 (1.16) | 0.50–6.16 | 1.41 (0.78) | 0.45–5.07 |
| NMFOL | Baseline | 5.33 (3.24) | 1.09–19.6 | 5.20 (3.20) | 0.81–17.4 | 5.57 (2.87) | 1.71–16.4 |
|  | Intermediate | 1.34 (0.94) | 0.47–6.46 | 1.94 (1.09) | 0.61–5.45 | 4.70 (2.15 | 0.96–11.7 |
|  | Endline | 1.35 (0.64) | 0.55–5.11 | 1.76 (0.98) | 0.57–5.27 | 4.58 (2.04) | 1.06–11.1 |

Abbreviations: 5-methylTHF, 5-methyltetrahydrofolate; HFS, higher folic acid iodized salt; IS, iodized salt with no added folic acid; LFS, lower folic acid iodized salt; MeFox, pyrazino-s-triazine derivative of 4α-hydroxy-5-methyltetrahydrofolate; NMFOL, non-methyl folate; TFOL, total folate; UMFA, unmetabolized folic acid.

^1^ Range of concentrations reflects minimum and maximum measured concentrations or percent of TFOL.

^2^ HFS fortified with ~99 ppm folic acid to provide an additional 600 ug folic acid/d); LFS fortified with ~33 ppm folic acid to provide an additional 200 ug folic acid/d; all salts fortified with ~32 ppm iodine as potassium iodate.

^3^ Intermediate timepoint randomly assigned between 4 and 20 wk; endline at 24 to 26 wk after initiating the intervention.

**Supplementary Table 2.** Spearman correlation between various serum folate biomarkers by intervention arm and timepoint

|  | **NMFOL^1^** | **UMFA** | **MeFox** | **THF** | **TFOL^2^** |
| --- | --- | --- | --- | --- | --- |
| **Baseline (all groups)** |  |  |  |  |  |
| 5-MethylTHF | 0.12* | 0.19* | 0.06 | 0.12* | 1.00* |
| NMFOL |  | 0.02 | 0.28* | 1.00* | 0.16* |
| UMFA |  |  | 0.00 | 0.02 | 0.20* |
| MeFox |  |  |  | 0.28* | 0.07 |
| THF |  |  |  |  | 0.16* |
| **Endline IS group** |  |  |  |  |  |
| 5-MethylTHF | 0.25* | 0.39* | 0.14 | 0.26* | 1.00* |
| NMFOL |  | 0.11 | 0.02 | 1.00* | 0.27* |
| UMFA |  |  | 0.42* | 0.11 | 0.40* |
| MeFox |  |  |  | 0.00 | 0.15 |
| THF |  |  |  |  | 0.28* |
| **Endline LFS group** |  |  |  |  |  |
| 5-MethylTHF | 0.03 | 0.26* | 0.23* | 0.03 | 1.00* |
| NMFOL |  | -0.05 | -0.12 | 1.00* | 0.04 |
| UMFA |  |  | 0.28* | -0.05 | 0.27* |
| MeFox |  |  |  | -0.12 | 0.23* |
| THF |  |  |  |  | 0.04 |
| **Endline HFS group** |  |  |  |  |  |
| 5-MethylTHF | 0.28* | 0.19 | 0.25* | 0.26* | 0.99* |
| NMFOL |  | 0.18 | 0.04 | 1.00* | 0.30* |
| UMFA |  |  | 0.29* | 0.16 | 0.24* |
| MeFox |  |  |  | 0.05 | 0.25* |
| THF |  |  |  |  | 0.29* |

Abbreviations: 5-methylTHF, 5-methyltetrahydrofolate; HFS, higher folic acid iodized salt; IS, iodized salt with no added folic acid; LFS, lower folic acid iodized salt; MeFox, pyrazino-s-triazine derivative of 4α-hydroxy-5-methyltetrahydrofolate; NMFOL, non-methyl folate; THF, tetrahydrofolate; TFOL, total folate; UMFA, unmetabolized folic acid.

^1^ NMFOL represents the sum of 3 minor folate forms: THF, 5-formyltetrahydrofolate, and 5,10-methenyltetrahydrofolate.

^2^ TFOL represents the sum of biologically active folate forms: 5-methylTHF, UMFA, and NMFOL (MeFox is not biologically active).

* Indicates significant correlation, *P* <0.05.

**Supplementary Table 3.** Response of serum or plasma folate forms to various folic acid intervention studies

| **Study (reference)** | **Timepoint** | **Intervention^1^** | **Serum/plasma folate form concentrations (nmol/L)^2^** | | | |
| --- | --- | --- | --- | --- | --- | --- |
|  |  | **(µg FA/d)** | **5-MethylTHF** | **UMFA** | **NMFOL** | **MeFox** |
| Ethiopia, LFS group^3^ | Baseline | n/a | 13.7 (10.2, 18.0) | 0.20 (0.10, 0.28) | 0.64 (0.46, 0.85) | 1.36 (0.92, 2.27) |
|  | After 6 mo | 277 (F) | 44.2 (34.4, 57.7) | 0.85 (0.72, 1.07) | 0.72 (0.46, 0.90) | 1.56 (1.23, 2.29) |
| U.S. NHANES (8)^4^ | Post-fortification | 100–200 (F) | 39.3 (26.1, 58.6) | 0.68 (0.51, 0.93) | 1.18 (0.90, 1.56) | 1.11 (0.75, 1.70) |
| Cameroon (7)^5^ | Post-fortification | 68 (F) | 31.3 (26.1, 42.2) | 0.69 (0.48, 1.01) | 0 (0, 0) | 6.6 (5.1, 8.9) |
| Honduras (4)^6^ | Baseline | n/a | 18.8 (5.6) | 0.62 (0.29) | No data | No data |
|  | At 12 wk | 1,000 (S) | 37.7 (10.6) | 2.28 (3.11) | No data | No data |
| China (2)^7^ | Baseline | n/a | 10.5 (5.95) | 0.35 (0.08) | No data | No data |
|  | At 6 mo | 100 (S) | 16.3 (7.21) | 0.53 (0.20) | No data | No data |
|  | Baseline | n/a | 12.5 (6.18) | <LOD | No data | No data |
|  | At 6 mo | 400 (S) | 25.8 (15.3) | 0.64 (0.26) | No data | No data |
|  | Baseline | n/a | 9.1 (4.73) | 0.30 | No data | No data |
|  | At 6 mo | 4,000 (S) | 27.3 (22.2) | 0.79 (0.39) | No data | No data |

Abbreviations: 5-methylTHF, 5-methyltetrahydrofolate; FA, folic acid; IQR, interquartile range; LFS, lower folic acid iodized salt; MeFox, pyrazino-s-triazine derivative of 4α-hydroxy-5-methyltetrahydrofolate; NMFOL, non-methyl folate; UMFA, unmetabolized folic acid.

^1^ Letter in parenthesis indicates whether the intervention was from fortified food (F) or a supplement (S).

^2^ Folate form concentrations are presented as median (IQR) or mean (SD); Cameroon: plasma; other studies: serum.

^3^ Ethiopia randomized intervention trial with double-fortified salt; fasting females 18**–**49 y (*n*=113).

^4^ U.S. NHANES 2011–2016, 15 y after introduction of enriched cereal grain fortification; fasting females ≥1 y (*n*=5,042).

^5^ Cameroon, Yaoundé and Douala, 1 y after introduction of fortified wheat flour; fasting females 15**–**49 y (*n*=59); testing of 1 fortified flour sample showed 2.84 mg FA/kg flour (compared to 5 mg FA/kg flour mandate); median usual flour intake in women was 24 g/d.

^6^ Honduras randomized double-blind supplementation trial with folic acid; fasting females 18**–**49 y (*n*=43).

^7^ China randomized double-blind supplementation trial with folic acid; fasting females 24**–**42 y (*n*=30 per arm).

**Supplementary Figure 1.** Study profile (**1**)

Contacted & screened

n=1287

Not using contraceptives, n=427

Refused screening, n=111

Hypertension, n=90

Pregnant, n=75

Intending to relocate, n=26

*Screening & exclusions*

Potentially eligible & consenting for initial blood draw

n=558

Not available, n=66

Refused exam, n=56

Inaccessible residence, n=12

Completed baseline exam & assigned study number

n=424

Stopped using contraceptives, n=25

Hypertension. n=19

Refused participation, n=14

Pregnant (n=3) or anemic (n=3)

*Enrolled in trial, n=360*

Group IS

n=122

Group LFS

n=113

Group HFS

n=125

Exited early, n=13

Refused, n=6

No contraception, n=6

Supplements. n=1

Exited early, n=5

Refused, n=2

Relocated, n=2

No contraception, n=1

Exited early, n=18

Refused, n=12

Relocated, n=3

No contraception, n=3

Completed study

n=109

Completed study

n=108

Completed study

n=107

**Reference**

1. M. Tessema, D. Nane, M. Woldeyohannes, I. Agbemafle, C.M. McDonald, C.D. Arnold, *et al.*, Folic acid fortification of iodized salt improves the folate status of nonpregnant adult Ethiopian females and does not impair their iodine status: a community-based, household-randomized, dose-response trial, Am. J. Clin. Nutr. 123 (2026) 101204, <https://doi.org/10.1016/j.ajcnut.2026.101204>.

**Supplementary Figure 2.** Distributions of initial (panels A-E) and final (panels F-J) concentrations of serum total folate and folate forms by intervention arm. The final timepoint was at 24 to 26 wk after initiating the intervention. The global *P*-value assesses overall differences across the intervention arms for each folate outcome variable and timepoint.

Abbreviations: 5-methylTHF, 5-methyltetrahydrofolate; HFS, higher folic acid iodized salt; IS, iodized salt with no added folic acid; LFS, lower folic acid iodized salt; MeFox, pyrazino-s-triazine derivative of 4α-hydroxy-5-methyltetrahydrofolate; UMFA, unmetabolized folic acid.

| **A**  **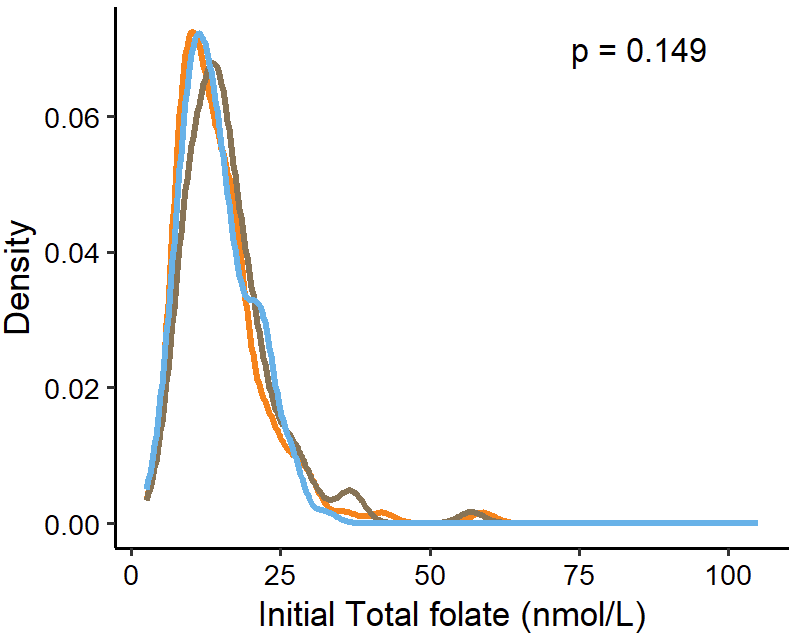** | **B**  **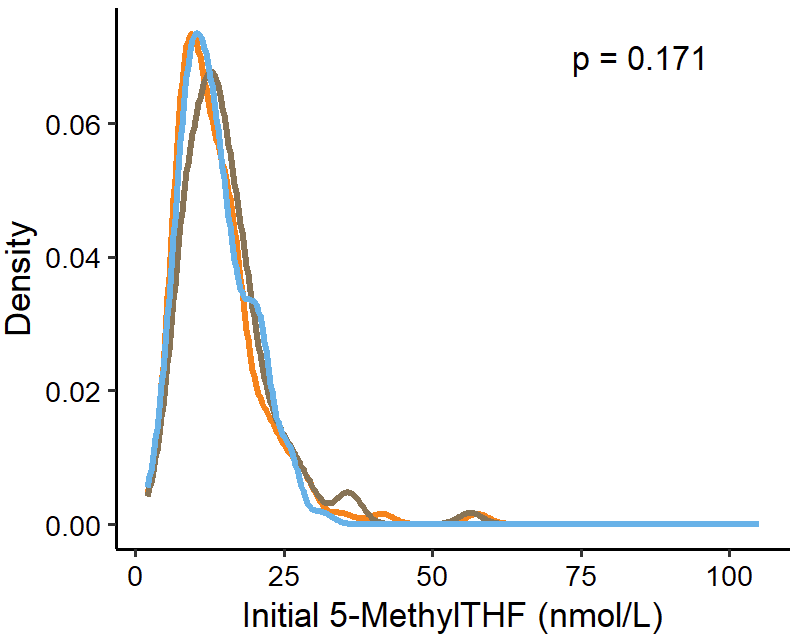** | **C**  **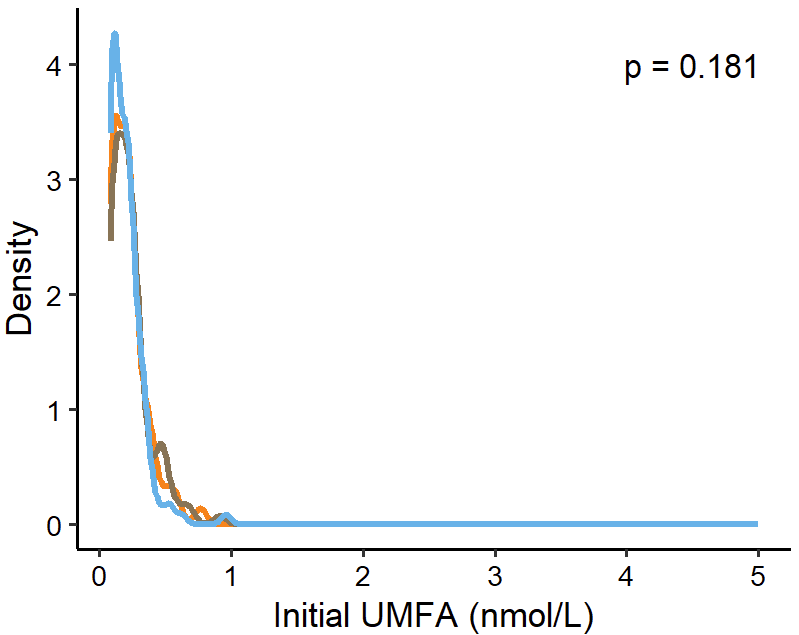** | **D**  **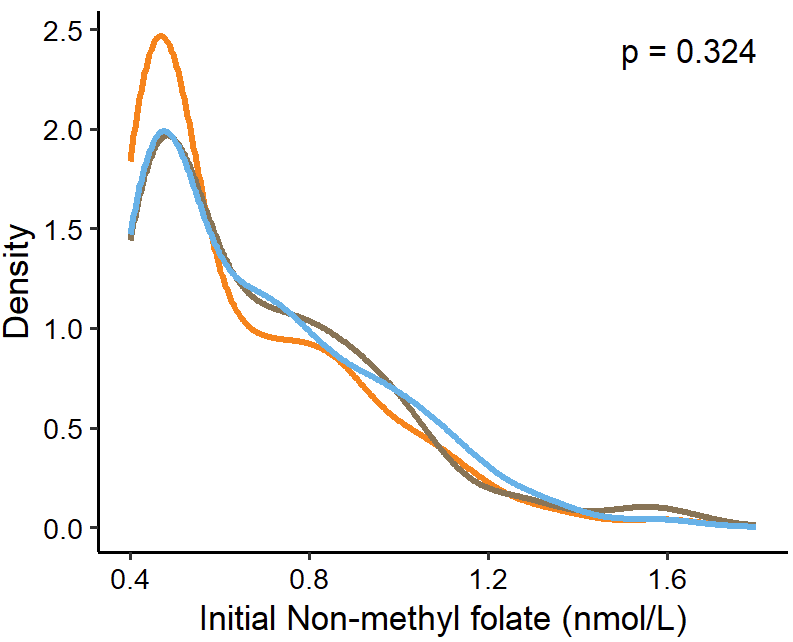** | **E**  **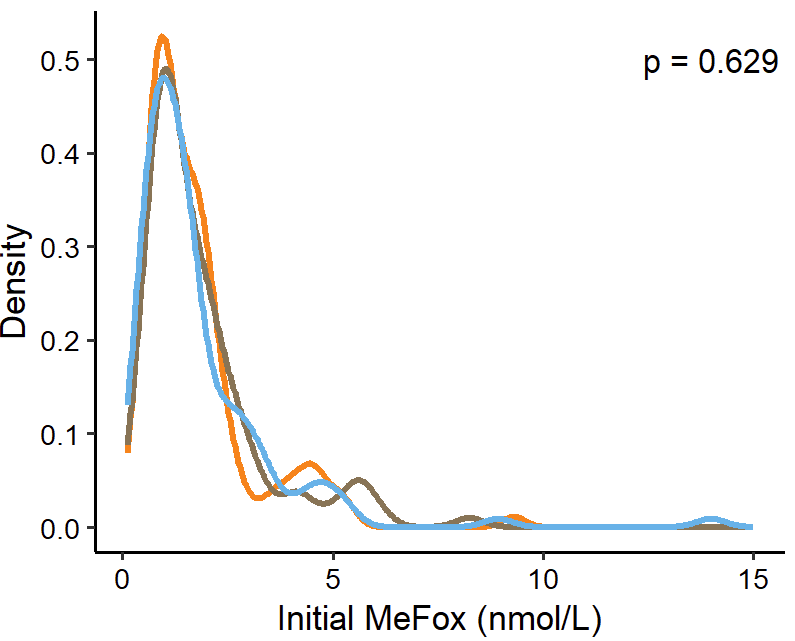** |
| --- | --- | --- | --- | --- |
| **F**  **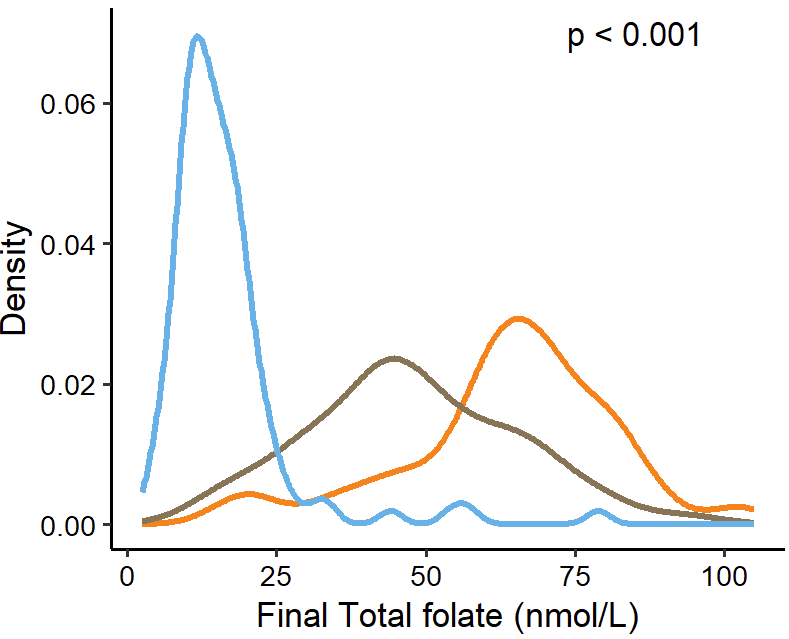** | **G**  **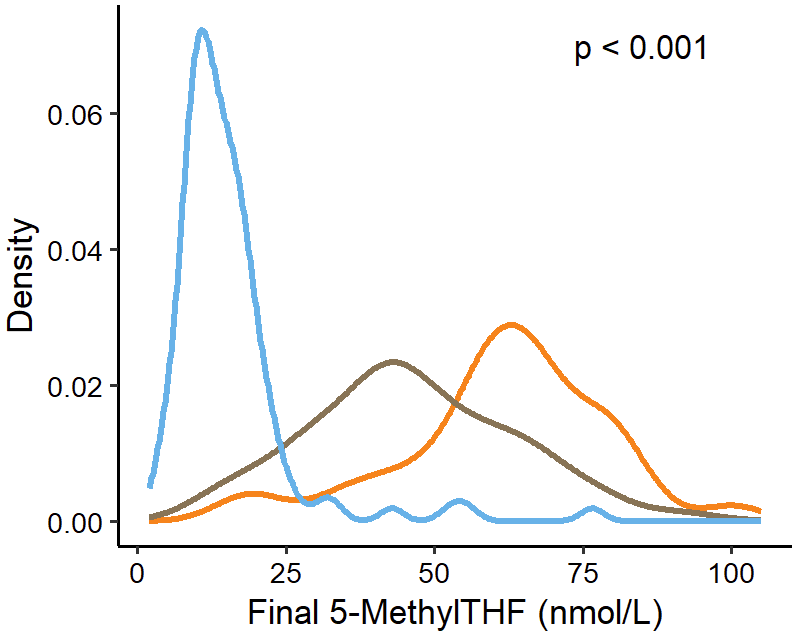** | **H**  **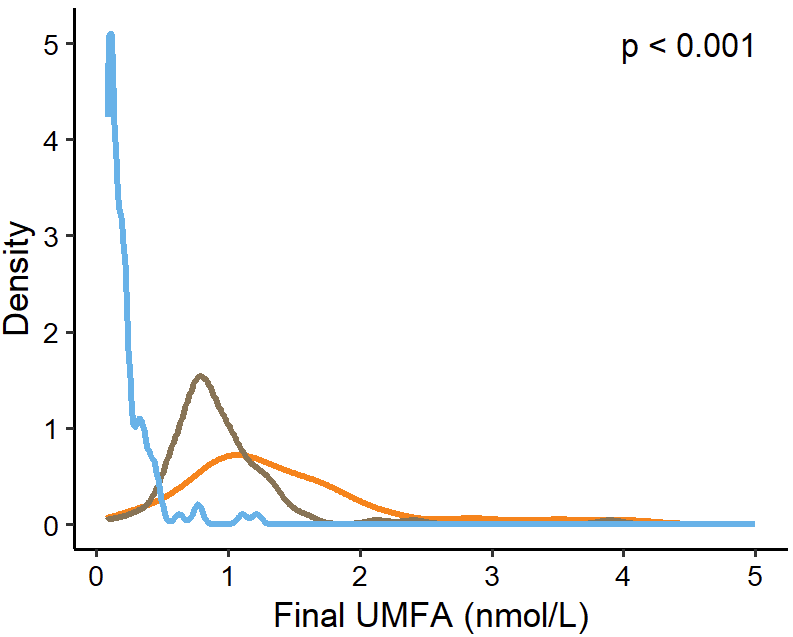** | **I**  **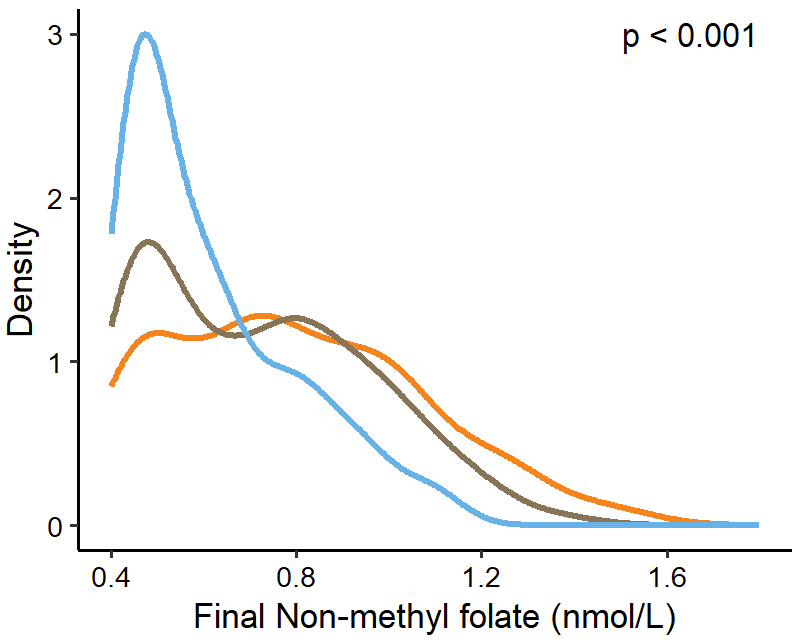** | **J**  **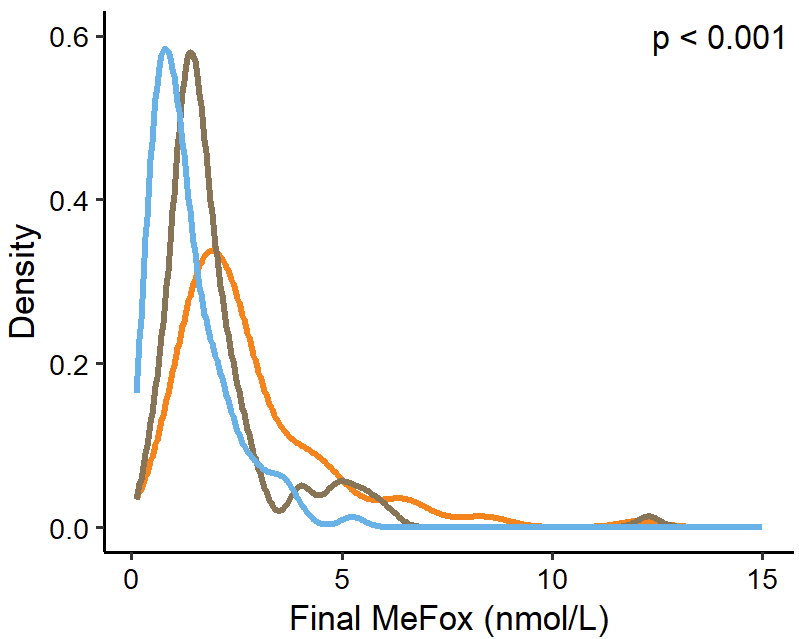** |

**
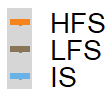
**
